# Wrist-ankle acupuncture (WAA) for precompetition nervous syndrome: study protocol for a randomized controlled trial

**DOI:** 10.1186/s13063-015-0910-z

**Published:** 2015-09-07

**Authors:** Shi Shu, Mei Zhan, Yan-li You, Xiao-lu Qian, Chun-ming Li, Cheng-lin Zhou, Shuang Zhou

**Affiliations:** Changhai Hospital of Traditional Chinese Medicine, Second Military Medical University, 168 Changhai Road, Yangpu District Shanghai, 200433 China; Department of Sport Psychology, School of Kinesiology, Shanghai University of Sport, 399, Changhai Road, Yangpu District Shanghai, 200433 China

**Keywords:** Wrist-ankle acupuncture (WAA), Precompetition nervous syndrome, Anxiety, Randomized controlled trial

## Abstract

**Background:**

Precompetition nervous syndrome comprises an excessive nervous and anxiety response to the high-pressure environment preceding a sporting competition. The use of acupuncture as a treatment option for anxiety, and wrist-ankle acupuncture (WAA) specifically in this instance, has been identified as a growing trend within the Western world. In our previous study, we have confirmed the efficacy of WAA for pre-examination anxiety. In this paper, we present a randomized controlled single-blind trial evaluating the use of WAA for precompetition nervous syndrome, comparing it with the intervention of sham acupuncture.

**Methods/Design:**

The study was designed as a randomized controlled single-blind trial to evaluate the effects of WAA for precompetition anxiety. The trial will be conducted in annual track and field events of Shanghai University of Sport. A total of 100 participants who meet inclusion criteria are randomly assigned by computerized randomization to receive WAA therapy or sham acupuncture. The group allocations and interventions are concealed to participants and statisticians. The Competition State Anxiety Scale (CSAI-2) is used as the primary outcome measure, while heart rate, blood pressure, respiratory frequency, tension syndrome curative effect evaluation and participants’ feeling of acupuncture questionnaire are applied as secondary outcome measures.

**Discussion:**

The results of this trial will confirm whether WAA is effective to treat precompetition anxiety in annual track and field events.

**Trial registration:**

Chinese Clinical Trial Registry (identifier: ChiCTR-TRC-13003931; registration date: 22 October 2013).

## Background

With unceasing improvement in the standard of modern sports at competitive level, the gap between the level of athletic technique and physical ability is ever diminishing. The results of the competition often depend not only on the gap between technique and physical ability, but are greatly influenced by the athletes’ psychological fitness. Thus, psychological factors have become an important component of performance in modern competitive sports.

Precompetition nervous syndrome comprises an excessive nervous and anxiety response to the high-pressure environment preceding a sporting competition. Although a certain level of anxiety enhances performance, uncontrolled emotions and negative cognitions can have adverse effects [1–3]. Various techniques have been used to help athletes control their emotions. These include self-breathing training, biofeedback training, meditation, visuomotor behavior rehearsal, hypnosis, and cognitive behavioral therapies [4]. The clinical trial evidence supporting the lasting effectiveness of these methods for athletes is limited and generally weak [5]. It is apparent that research is greatly needed in this area. Moreover, it is apparent that a robust intervention to consistently control performance anxiety is also critically needed.

Acupuncture has been used worldwide for various types of disorders of spirit and emotion, such as depression, anxiety and insomnia [4–10]. In some international sporting events, like the 2010 Asian Games of Guangzhou, a South Korean player received acupuncture when competing with her rival at the game of *Go* in order to help her to keep relaxed and calm. This suggests that using acupuncture in sports is feasible for players’ mental preparation. On the other hand, traditional acupuncture treatments emphasize the “needling sensation”, which will give an unpleasant feeling to some players. Knowledge of these effects has impeded the wider acceptance of traditional acupuncture treatments in the games. Wrist-ankle acupuncture (WAA) is a modern acupuncture technique invented and developed by Professor Zhang Xinshu and his colleagues from Changhai Hospital of Second Military Medical University, Shanghai, China in the 1970s [11]. The theory of WAA has a unique system of its own, which is quite different from that of traditional acupuncture [12]. WAA does not produce the “needling sensation” and has demonstrated good effects for mental and nervous disorders. More importantly, the lack of pain and “needling sensation” during the performance of WAA imposes minimal psychological stress on the player and hence is readily accepted.

Previously, we have confirmed the efficacy of WAA for pre-examination anxiety [13]. In the study we observed that WAA can significantly alleviate exam tension and that the treatment is very safe. However, to date, there are no studies investigating the application of WAA for managing precompetition anxiety in athletes. The present study attempts to investigate the effects of WAA for athletes with precompetition anxiety in annual track and field events.

The trial protocol here is in accordance with Consolidated Standards of Reporting Trials (CONSORT) 2010 statement [14]. The intervention WAA details are described in accordance with the STandards for Reporting Interventions in Clinical Trials of Acupuncture (STRICTA) 2010 extension [15].

## Methods/Design

### Ethics review and informed consent

The study protocol was reviewed and approved by the Chinese Ethics Committee of Registering Clinical Trials (ChiECRCT-2013024), and subsequently in the Chinese Clinical Trial Registry (ChiCTR-TRC-13003931). All participants are required to provide informed consent.

### Study design

**Fig. 1:**
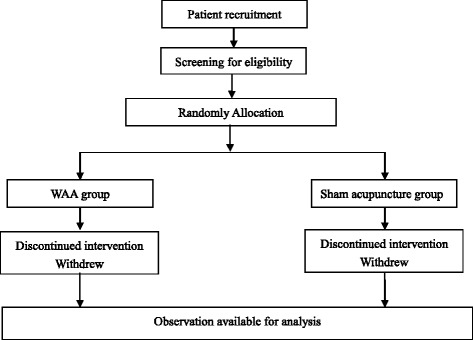
Flowchart of the study design

The study was designed as a randomized controlled single-blind trial to evaluate the effects of WAA for precompetition anxiety (Fig. 1). The trial will be conducted in annual track and field events of Shanghai University of Sport.

### Study procedure and patient recruitment

The trial information leaflets will be distributed to the university campus to recruit potential participants 1 week before the competition. The leaflets contain a brief questionnaire for screening participants with precompetition nervous syndrome. Those who have two or more major symptoms of excessive nervous and anxious, insomnia, fear of failure, and inattention are recruitable. One item in the questionnaire is to distinguish whether these symptoms are responses that are present before the competition [16]. Participants are included only if they meet the inclusion criteria and sign an informed consent document. The protocol details are also explained to the participants in the leaflets and all the basic information will be provided to the researcher.

### Key inclusion and exclusion criteria

#### Inclusion criteria

1. Sport university students, age 18 to 40 years old, both male and female participants;
2. The patient must not yet have previously received the WAA treatment;
3. The patient must have signed the informed consent.

#### Exclusion criteria

1. Precompetition nervous syndrome participants using drugs or other concurrent systems;
2. A history of mental illness or physical disease;
3. A depressive trend (depression self-rating scale score ≧ 41).

### Data collection

All the participants will be asked to complete two identical scale questionnaires and physical condition examinations before and after the treatment in order to assess their anxiety levels. The first questionnaire and physical condition examination must be completed 30 minutes before the treatment, while the second one will be collected 30 minutes after the treatment.

### Treatment regimen

Participants are required to receive 1 treatment 3 hours before the track event sessions to minimize uncertainty in the selection of different events (only track event athletes are included as subjects).

### Interventions

**Fig. 2:**
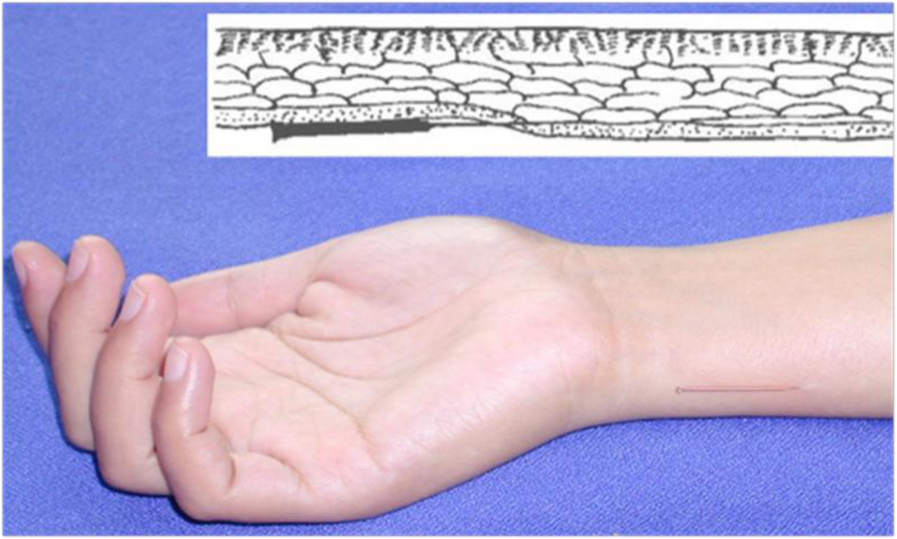
Wrist-ankle acupuncture (WAA) needling point 1 on the wrist zone (point upper 1) after insertion. Point upper 1 is located on the pit between the medial border of the ulnar and the tendon of musculus flexor carpi ulnaris

The treatment scheme originates from the principles of WAA and has been practiced in previous clinical trials for several decades [11–13, 17–20]. According to the theory of WAA, each side of the body and each limb are longitudinally divided into six zones and one needling point is defined in each zone at the wrist or ankle. Needling point 1 on the wrist zone (point upper 1) can relieve disorders of emotion. For participants in the current study, precompetition anxiety belongs to disorders of emotion; therefore, all subjects were needled at point 1 at both wrists (Fig. 2).

WAA group: WAA was administered on point upper 1 at both wrists, 3 hours before the track event sessions. The needle was retained for 30 minutes. The subjects were asked to stay in the sitting position and to wear an eye mask. The target point was disinfected with an iodophor disinfectant (Shanghai Likang Disinfectant Hi-tech Co. Ltd., Shanghai, China). The processed needles were also held with three right-hand fingers (thumb, index finger, and middle finger). The skin near the target point was gently pressed with the left thumb to make it slightly taut. A disposable sterile WAA needle (0.25 mm in diameter and 25 mm in length, Suzhou Medical Appliance Factory, Jiangsu Province, China) was held with three right-hand fingers (thumb, index finger, and middle finger). Then, the needle tip was swiftly inserted into the skin at the target point at a 30° angle. The needle was lowered to the horizontal position and slowly advanced until the entire needle (except the handle) entered the subcutaneous tissue. The handle was then fixed to the skin with an adhesive tape. The needles were retained in the subcutaneous tissue for 30 minutes. For a successful WAA treatment, the patient will only feel a negligible stabbing pain when the tip of the needle pierces the skin. No other needling sensation will be experienced. A single registered acupuncturist, with at least 1 year of previous WAA experience, administered the care to all subjects [11, 17].

**Fig. 3:**
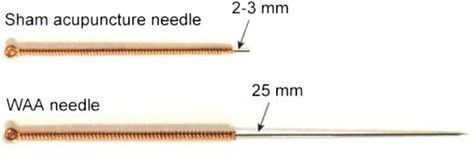
Wrist-ankle acupuncture (WAA) needle and sham acupuncture needle. The WAA needle is a disposable sterile needle (0.25 mm in diameter and 25 mm in length, Suzhou Medical Appliance Factory, Jiangsu Province, China); the sham acupuncture also uses the same model needle (0.25 mm in diameter and 25 mm in length, Suzhou Medical Appliance Factory, Jiangsu Province, China), although the tip and most of the body was cut off and blunted. Only a 2–3 mm length of the needle body remained

Sham acupuncture group: sham acupuncture was administered on point 1 at the wrists, 3 hours before the track event sessions. The needle was retained for 30 minutes. The subjects were asked to stay in the sitting position and to wear an eye mask. The target site was also disinfected with an iodophor disinfectant (Shanghai Likang Disinfectant Hi-tech Co. Ltd., Shanghai, China). The tip and most of the body of the disposable sterile acupuncture needle (0.25 mm in diameter and 25 mm in length, Suzhou Medical Appliance Factory, Jiangsu Province, China) (Fig. 3) was cut off and blunted. Only 2–3 mm of the length of the needle body remained. The processed needles were also held with three right-hand fingers (thumb, index finger, and middle finger). The skin near the target site was gently pressed with the left thumb to make it slightly taut. The needle tip swiftly punctured the skin at the target point at a30° angle (the tip was not actually inserted into the skin). Then, the needles remained horizontally on the skin of the point for 30 minutes. The handle was also fixed to the skin with adhesive tape. For a successful sham treatment, the patient will also only feel a negligible stabbing pain when the tip of the needle pierces the skin. No other needling sensation will be experienced. As with the WAA group, a single registered acupuncturist with at least 1 year of previous WAA experience, administered the care to all subjects.

### Randomization and blinding

An outside researcher who is not allowed to directly contact with the participants will perform computerized randomization. The assessor will also be blinded to the treatment allocation. The acupuncturist performing the WAA intervention and sham WAA intervention cannot be completely blinded, but he is not be allowed to reveal any information about treatment procedures and outcomes to the participants or the assessor. Thus, both the participants and the assessor will not distinguish clearly which intervention has been given. A sealed envelope containing an allocation sequence number for each participant will be opened after each participant meets eligibility criteria and informed consent is obtained. If any error or disclosure with regard to randomization occurs, a new randomization sequence will be generated, starting from the problematic serial number, and subsequently applied to the participant.

### Sample size calculation and statistical analysis

The sample size is estimated, based on changes of Competition State Anxiety Scale (CSAI-2) before and after treatment, from a pilot study. The pilot study was a two-arm design with WAA group and sham acupuncture group. The means of changes of WAA group and sham acupuncture group are 1.9 and −2.0 respectively, while the standard deviations are 6.9 and 5.5. The software Power Analysis and Sample Size (SAS version 9.3, SAS Institute Inc., Cary, NC, USA) was used to perform the sample size calculation. The sample size was calculated with a significance level of 0.05 and power of 0.80. The result was a total required sample size of 86, with 43 for each group. With a maximum dropout tolerance of 15 %, 7 participants are needed for each group. Therefore, 100 participants are needed for the trial, with 50 for each group.

Statistical analysis will be performed using SPSS 15.0 statistics software (SPSS Inc., Chicago, IL, USA) and the statistician is blinded from group allocation. For quantitative data, the distribution pattern and homogeneity of variance will be examined. If there is a symmetric distribution, the mean (M) ± standard deviation will be used for statistical description. A paired samples *t* test will be examined to compare the differences between the quantitative indices before and after treatment in one group, while an independent sample *t* test will be used for comparison between groups. The entire statistical test will use bilateral examination where *P* < 0.05 indicates statistically significant difference.

### Primary outcome

#### Competition State Anxiety Scale (CSAI-2)

The CSAI-2 questionnaire is compiled by Martens et al. [21] on the theory of multidimensional competition state anxiety. The revision of CSAI-2 was re-corrected by ZhuBeili (1994) [22]. The scale is composed of 3 subscales: each subscale score, respectively, has 9 items. Scores are from 9 to 36, the higher the score means the higher the cognitive state anxiety, the somatic state anxiety and the state self-confidence. It is the primary outcome to assess competitive anxiety and the efficacy of treatment.

### Secondary outcomes

Heart rate, blood pressure, respiratory frequency, tension syndrome curative effect evaluation, and participants’ feeling of acupuncture questionnaire will also be rated.

### Data and safety monitoring

The data will be collected by a well-trained assessor to improve data quality and regular monitoring will be conducted for quality control by a researcher. The assessment of safety will primarily be based on the frequency of adverse events (AEs). AEs will be defined as any unfavorable signs or symptoms temporally associated with WAA treatment, such as subcutaneous bleeding, fainting, heart palpitations, dizziness during acupuncture treatment. The severity and causality of AEs will be immediately recorded in the case report form by the researcher, who will report the AE to the primary investigator and ethics committee. Then, the decision will be made on whether the patient needs to withdraw from the trial. If any AEs occur, the appropriate treatment will be immediately provided to the participant.

## Discussion

To our knowledge, the use of the common filiform needle in acupuncture intervention is difficult to incorporate into a blind trial because of the special intervention procedure. According to traditional acupuncture theory, filiform needle therapy to the acupoints always gives the participants the sensation of obtaining *qi* (eg. sourness, numbness, distention and pain). An acupuncture trial design without requesting whether *qi* is obtained by the participants could not reflect the correct and real therapeutic effect of acupuncture [23]. However, for WAA, needling sensation is not required. It is innovative that we could design a randomized controlled single-blind trial with a non-penetrating sham WAA group. In the trial, the subjects were asked to wear an eye mask. Under this condition, they did not know which intervention they would be given and also could not distinguish therapuetic intervention in the subsequent treatment. The participants’ feeling of acupuncture questionnaire would be arranged at the end of treatment in order to exclude the breaking of blinded subjects. Therefore, we believe that this method can serve as an effective placebo intervention and that this study will be able to provide high-quality evidence for the efficacy of WAA for precompetition nervous syndrome.

The trial also has a limitation. The degree to which precompetition nervous syndrome is experienced is closely related to the championship level at which sport is played. Hence, higher levels of competition will cause more precompetition anxiety. Therefore, the trial may be more easily performed at this level as it will provide a greater number of participants. In this study, we could not have conducted the trial in large national sporting events due to the limits imposed by those conditions. Thus, the trial will only reflect the treatment effects in certain circumstances, but hopefully the results will yield important information to provide help and useful advice to the athletes in national athletic meetings or at future Olympic Games.

## Trial status

Participants are currently being recruited into the study. The study will be finished on 30 September 2015.

### Consent

Written informed consent was obtained from the patient for publication of this manuscript and accompanying the image in Fig. 2. A copy of the written consent is available for review by the editor-in-chief of this journal.

## Abbreviations

AEs: Adverse events
CONSORT: Consolidated standards of reporting trials
CSAI-2: Competition state anxiety scale
M: Mean
STRICTA: Standards for reporting interventions in clinical trials of acupuncture
WAA: Wrist-ankle acupuncture
